# Large language models for automated and audience-tailored labeling of latent classes

**DOI:** 10.1093/jamiaopen/ooag058

**Published:** 2026-04-28

**Authors:** Fatemeh Gholi Zadeh Kharrat, Rob Werfelmann, Glen Ep Ropella, Wolf Mehling, C Anthony Hunt, Jeffrey Lotz, Thomas A Peterson, REACH Investigators, Zehra Akkaya, Prakruthi Amar Kumar, Jeannie Bailey, Julia Barylak, Sigurd Berven, Andrew Bishara, Dennis M Black, Noah Bonnheim, Atul Butte, Joel Castellanos, Jennifer Cummings, Karina Del Rosario, Emilia Demarchis, Sibel Demir-Deviren, Susan K Ewing, Adam R Ferguson, Aaron Fields, Scott M Fishman, Sergio Garcia Guerra, Fatemeh Gholi Zadeh Kharrat, Xiaojie (Summer) Guo, Misung Han, Trisha Hue, J Russell Huie, C Anthony Hunt, Anastasia Keller, Karim Khattab, Roland Krug, Gregorji Kurillo, Feng Lin, Thomas Link, Jeffrey Lotz, John LynchTong Lyu, Rob Matthew, Wolf Mehling, Esmeralda Mendoza, Praveen Mummaneni, Caroline Navy, Conor O’Neill, Jessica Ornowski, Thomas Peterson, Ananya Rupanagunta, Aaron Scheffler, Shalini Shah, Irina Strigo, Naoki Takegami, Abel Torres-Espin, Salvatore Torrisi, Sachin Umrao, Rohit Vashisht, Joanna Veres, An (Joseph) Vu, Mark Steven Wallace, Lucy Ann Wu, Po-Hung Wu, Fadel Zeidan, Patricia Zheng, Jiamin Zhou

**Affiliations:** Department of Orthopaedic Surgery, University of California, San Francisco, CA 94143, United States; Bakar Computational Health Sciences Institute, University of California, San Francisco, CA 94158, United States; Tempus Dictum, Inc, Olympia, WA 98502, United States; Tempus Dictum, Inc, Olympia, WA 98502, United States; Department of Family and Community Medicine, Osher Center for Integrative Health, University of California, San Francisco, CA 94115, United States; Department of Bioengineering and Therapeutic Sciences, University of California, San Francisco, CA 94143, United States; Department of Orthopaedic Surgery, University of California, San Francisco, CA 94143, United States; Department of Orthopaedic Surgery, University of California, San Francisco, CA 94143, United States; Bakar Computational Health Sciences Institute, University of California, San Francisco, CA 94158, United States

**Keywords:** Large language models (LLMs), retrieval-augmented generation (RAG), orthopedic surgery, artificial intelligence (AI), LLaMA-3, LangChain, chronic low back pain (cLBP), Latent Class Model (LCM)

## Abstract

**Objective:**

This study compares multiple LLMs, including ChatGPT, DeepSeek, and Llama, to generate meaningful, audience-adapted labels for the existing latent classes among patients with chronic low back pain (cLBP).

**Methods:**

Phenotypes were derived from baseline data from two cohorts within the NIH HEAL BACPAC consortium: BACKHOME, a large nationwide e-cohort (train set: *N* = 3025), and COMEBACK, a deep phenotyping cohort (test set: *N* = 450). The analysis included pain characteristics, psychosocial factors, lifestyle habits, and social determinants of health. ChatGPT-4o (OpenAI), DeepSeek-R1, and Llama 3.3 (Meta) were applied to generate class labels for each combination of audience (clinician, patient, and caregiver), tone (formal, empathetic, and informal), and technicality (high, medium, and low).

**Results:**

Latent Class Model (LCM) identified four distinct behavioral phenotypes in patients with cLBP: *High Distress and Maladaptive Behaviors*, *Resilient and Adaptive Coping*, *Intermediate Maladaptive Patterns*, and *Emotionally Regulated with High Pain Burden*. Previously validated by domain experts, these profiles served as the basis for automated labeling using three LLMs (ChatGPT-4o, DeepSeek-R1, and Llama 3.3). Using different tones and complexity levels, each model produced class labels specific to clinicians, patients, and caregivers. The generated class names for all LLMs closely matched expert-defined traits like *emotional regulation*, *resilience*, and *high distress*, indicating strong conceptual alignment and the capacity of LLMs to generate precise, audience-specific labels for intricate behavioral and psychological profiles.

**Conclusions:**

These results highlight the possibility of integrating LLM-driven labeling into research and clinical practice, helping to achieve more transparent knowledge translation, improved decision-making, and personalized care.

## Introduction

Latent Class Model (LCM) is a statistical method that can be applied to various fields, including education, social sciences, and healthcare.^1–3^ LCM contains both Latent Class Analysis (LCA) and Latent Profile Analysis (LPA). It includes a mix of binary and continuous variables and techniques that aim to recover hidden groups from observed data.^2^^,^^4–6^ LCM models use observed indicators to infer these latent groups and determine their characteristic variable patterns, assuming that the observed variable distribution results from a finite mixture of underlying, unobserved subgroups.^2^ For example, Obbarius et al^7^ found four subgroups based on pain burden and emotional distress in patients undergoing multimodal inpatient treatment. Rovner et al^8^ determined four patient clusters based on pain acceptance levels, which were associated with specific patterns of psychological and physical function.

However, naming LCM-identified subgroups or clusters is a crucial step in making the findings interpretable and actionable. This often requires time consuming expert consensus, particularly when the LCM variables span a broad range of technical and clinical disciplines. Additionally, cluster names should be customized to the audience, as names suitable for clinicians may include language or jargon that is not interpretable or can even be offensive to patients.

Artificial intelligence (AI) has rapidly appeared as a transformative technology across numerous domains, including healthcare.^5^^,^^9–12^ Large language models (LLMs), which employ transformer architectures and encode rich contextual information using billions of parameters, are one example of these developments that have made it possible to understand and apply varied topics with nuance.^13^^,^^14^ Because LLMs are better at interpreting unstructured text than traditional AI models, which frequently rely on structured data and preset algorithms, they are invaluable for real-time data interpretation, supporting clinical decision-making, and improving patient engagement in clinical settings. LLMs such as ChatGPT,^15^ DeepSeek,^16^ and Llama^17^ can process and generate human-like language, making them valuable tools in various medical applications. ChatGPT, built on OpenAI’s GPT architecture, is available in multiple configurations; for example, GPT-4o^18^ contains approximately 200 billion parameters, while GPT-4o-mini has around 8 billion parameters. DeepSeek is an open-source language model with up to 671 billion parameters and context lengths of up to 128K tokens. Llama 3^19^ is available in different sizes, including 8 billion and 70 billion parameter versions.

LLMs are increasingly embraced for various healthcare applications, including orthopedic surgery, by enhancing diagnostic accuracy, streamlining administrative tasks, and improving patient care and education. For instance, Bains et al^20^ investigated the utility of LLMs, specifically ChatGPT, in providing patient information after total knee arthroplasty (TKA) and compared its performance with that of arthroplasty-trained nurses. Lieu et al^21^ examined the accuracy and comprehensibility of ChatGPT (Version 3.5) responses to frequently asked questions about scoliosis in a pediatric orthopedic context. Margetts et al^22^ examined the role of AI in scientific writing related to Alzheimer’s disease, osteoporosis, and fracture. Guillen et al^23^ investigated the utility of LLMs (ChatGPT, Llama 3.3, Microsoft Copilot) for patient Queries on Total Knee Replacement (TKR). Caterson et al^24^ demonstrated that LLMs could generate clinical letters and predict management plans for orthopedic scenarios. Sakai et al^25^ used GPT-4 Turbo for multi-label classification of inpatient comments, testing prompt strategies like zero-shot, in-context, and chain-of-thought. GPT-4 Turbo outperformed traditional and pre-trained models, offering an efficient way for healthcare providers to analyze patient feedback and improve responses. Wang et al^26^ showed that LLMs can be considered as implicitly inferring a hidden or “latent” variable carrying important task information, allowing for more accurate and contextually appropriate labeling, particularly in through in-context learning approaches.

LLMs and LCM aim to uncover meaningful patterns and structure within complex datasets, LLMs through contextually adaptive, language-based labeling, and LCM through statistical modeling of latent subgroups. In this current study, we combine the statistical rigor of LCM with the adaptive language capabilities of LLMs to enhance the interpretability and accessibility of class labeling in health research. We build upon our previously established LCM^27^ and leverage multiple LLMs (ChatGPT-4o, DeepSeek-R1, and Llama 3.3) to generate meaningful, audience-adapted labels (such as clinicians, patients, and caregivers) for these existing latent classes. Our results demonstrate the feasibility and advantages of integrating state-of-the-art LLMs with established statistical modeling, facilitating scientific rigor and effective communication in health research.

## Methods

### Phase 1: Latent class model development and characterization

We used LCM previously established in our earlier research within the NIH HEAL BACPAC consortium for this analysis. The BACKHOME,^28^ a large nationwide e-cohort (*N *= 3025) utilized for model training, and COMEBACK,^28^ as an external test set, a deep phenotyping cohort (*N *= 450) used for generalization. The datasets included a broad range of variables across several categories (for detailed descriptions, see Supplementary Appendix A): demographic factors (age, sex), anthropometrics (Body Mass Index [BMI]), pain-related measures (PEG score, PROMIS Pain Interference), pain characteristics (duration, frequency, intensity), and quality of life (PROMIS Physical Functioning [SF 6b]). Additional variables included mood indicators (PROMIS Depression, Pain Anxiety Symptoms [PAS]), pain persistence parameters (fear avoidance via FABQ-P, pain catastrophizing via PCS-6, pain self-efficacy via PSEQ-4), and elements of interoceptive awareness (Non-Distracting, Emotional Awareness, Self-Regulation) measured using the MAIA-2.

LCM was developed using model selection criteria that balance statistical fit, clinical interpretability, and parsimony. To determine the optimal number of classes, we employed 10-fold cross-validation with 100 bootstraps and evaluated model fit using Akaike Information Criterion (AIC), Bayesian Information Criterion (BIC), and entropy (uncertainty) to optimize model fit.^29^ AIC and BIC were used to balance fit and complexity, while entropy (uncertainty) measured the separation of latent classes. Evaluation of models ranging from 1 to 10 classes revealed that AIC and BIC values declined steeply from 1 to 4 classes, then plateaued, indicating that additional classes beyond 4 did not substantially improve model fit relative to increased complexity. Entropy values of 0.82 for BACKHOME (train) and 0.81 for COMEBACK (test) indicated well-separated, highly distinct latent classes. Furthermore, the four-class solution demonstrated strong clinical interpretability and adequate sample sizes within each class (BACKHOME: Class 1, *n* = 701; Class 2, *n* = 413; Class 3, *n* = 893; Class 4, *n* = 947), ensuring stable parameter estimates and statistical power. The resulting model was robust and generalizable, as evidenced by consistent fit metrics and class structure when applied to the independent COMEBACK test set. A detailed description of these models and their development is available elsewhere.^27^

After model estimation, the class-specific profiles of normalized indicator mean for four latent classes were obtained for both the BACKHOME (train set) and COMEBACK (test set) cohorts (see Supplementary Appendix B). The LCM was generated from extensive behavioral and clinical datasets, and all model outputs, class profiles, and variable definitions were methodically saved in DOCX format for use as input in the following stage, which involved automated class labeling with LLMs.

### Phase 2: Automated class labeling using large language models

#### Input preparation and standardization

This standardized input structure was provided identically to all three LLMs (ChatGPT-4o, DeepSeek-R1, and Llama 3.3) to ensure consistent information was provided to each model. Specifically, all three models received, identical variable definitions and scoring guidance, and (see Supplementary Appendix A), identical class profiles (see Supplementary Appendix B), and identical instructions specifying the audience, tone, and technicality parameters for label generation. Model-specific implementation parameters were optimized for each system’s architecture and access method, but these differences do not impact the comparability of outputs.

The standardized input and consistent temperature = 0.0 setting across all models (ChatGPT-4o, DeepSeek-R1, and Llama 3.3) means that any differences in the generated labels can be attributed to the models’ underlying architectures, training data, and inherent stylistic preferences, rather than to discrepancies in the input information or parameter settings.

#### Chain-of-thought prompting with ChatGPT and DeepSeek for automated latent

Chain-of-Thought (CoT)^30^ prompting is a natural language prompt engineering strategy used with LLMs to enhance their reasoning capabilities. Rather than simply instructing the model to provide an answer, CoT prompting explicitly guides the model to break down its reasoning into logical, interpretable steps before arriving at a final output. By encouraging the model to reveal its intermediary thought processes, this method can enhance its output’s caliber, interpretability, and dependability.^31^^,^^32^ For generating audience-tailored clinical labels from complex latent classes, where labels must simultaneously serve multiple stakeholders (clinicians, patients, caregivers) and satisfy competing requirements (clinical accuracy, accessibility, consistency), CoT offers different advantages. By requiring systematic reasoning about latent class features before label generation, CoT ensures that outputs are interpretable and justifiable to clinical users, logically consistent across audience contexts, and semantically accurate and clinically relevant. We therefore selected CoT prompting to conduct ChatGPT-4o and DeepSeek-R1 in generating audience-tailored class labels and asked the LLMs to generate label with justifications, outputting results in a standardized JSON format.

#### Automated latent class labeling with llama 3.3

Prompt formatting and response parsing were implemented using LangChain’s PromptTemplate and StrOutputParser.^33^ The Llama 3.3 model was accessed via the OllamaLLM interface and generated labels in a standardized JSON format.

### Phase 3: Model execution, output, and evaluation

Each output included narrative descriptions summarizing class profile distributions and a set of candidate class labels generated by the LLMs for each latent class. The experimental design tailored the suggested class names to different audiences, tones, and technicality levels. Experts thoroughly examined each set of narrative descriptions and class labels for interpretability, contextual appropriateness, and clarity in clinical practice and health communication. This assessment considered not only the accuracy and relevance of the labels, but also their potential utility for diverse stakeholders, including clinicians, patients, and caregivers.

**Figure 1:**
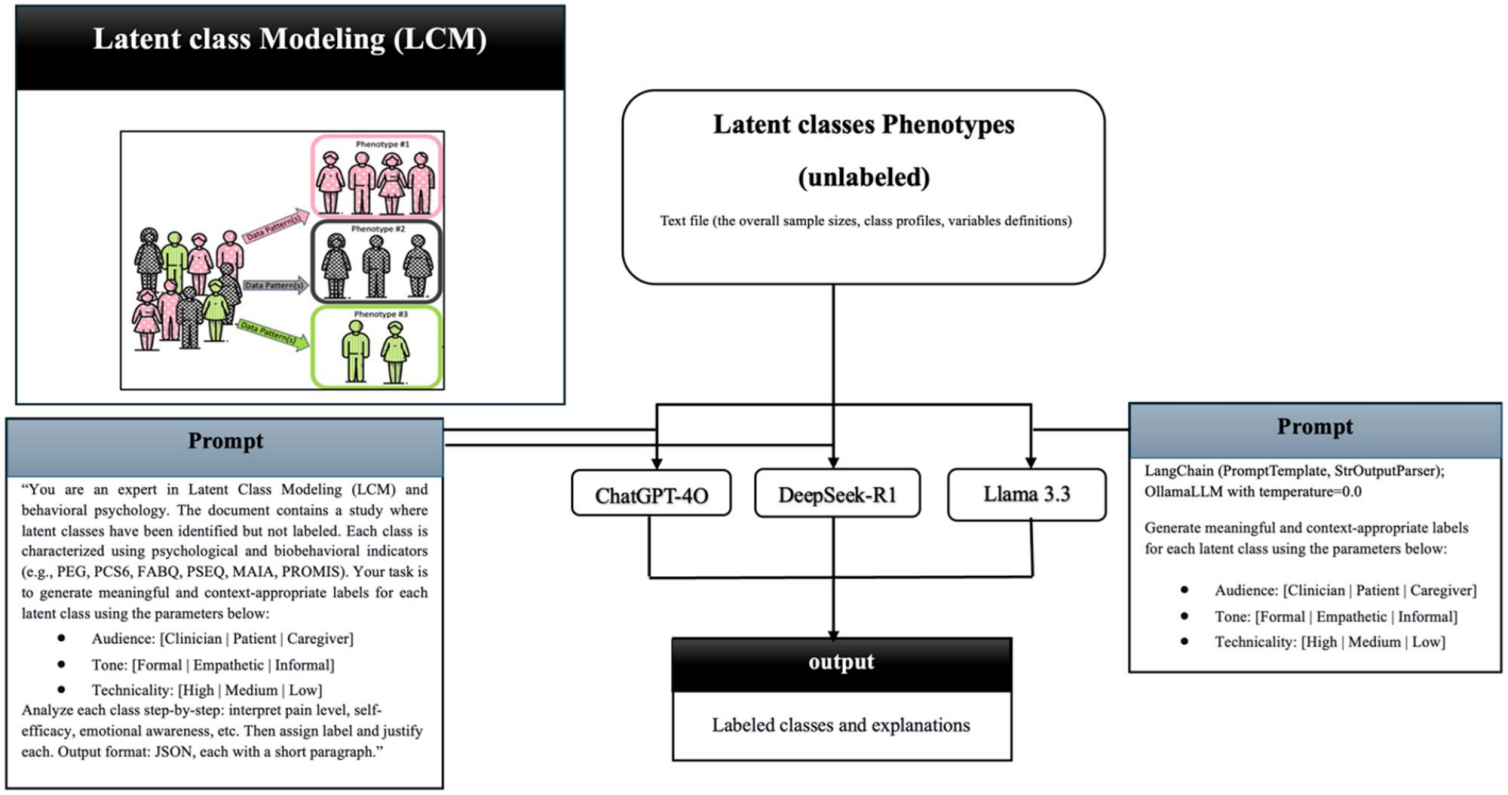
Schematic representation of the LLMs model.

**Figure 2:**
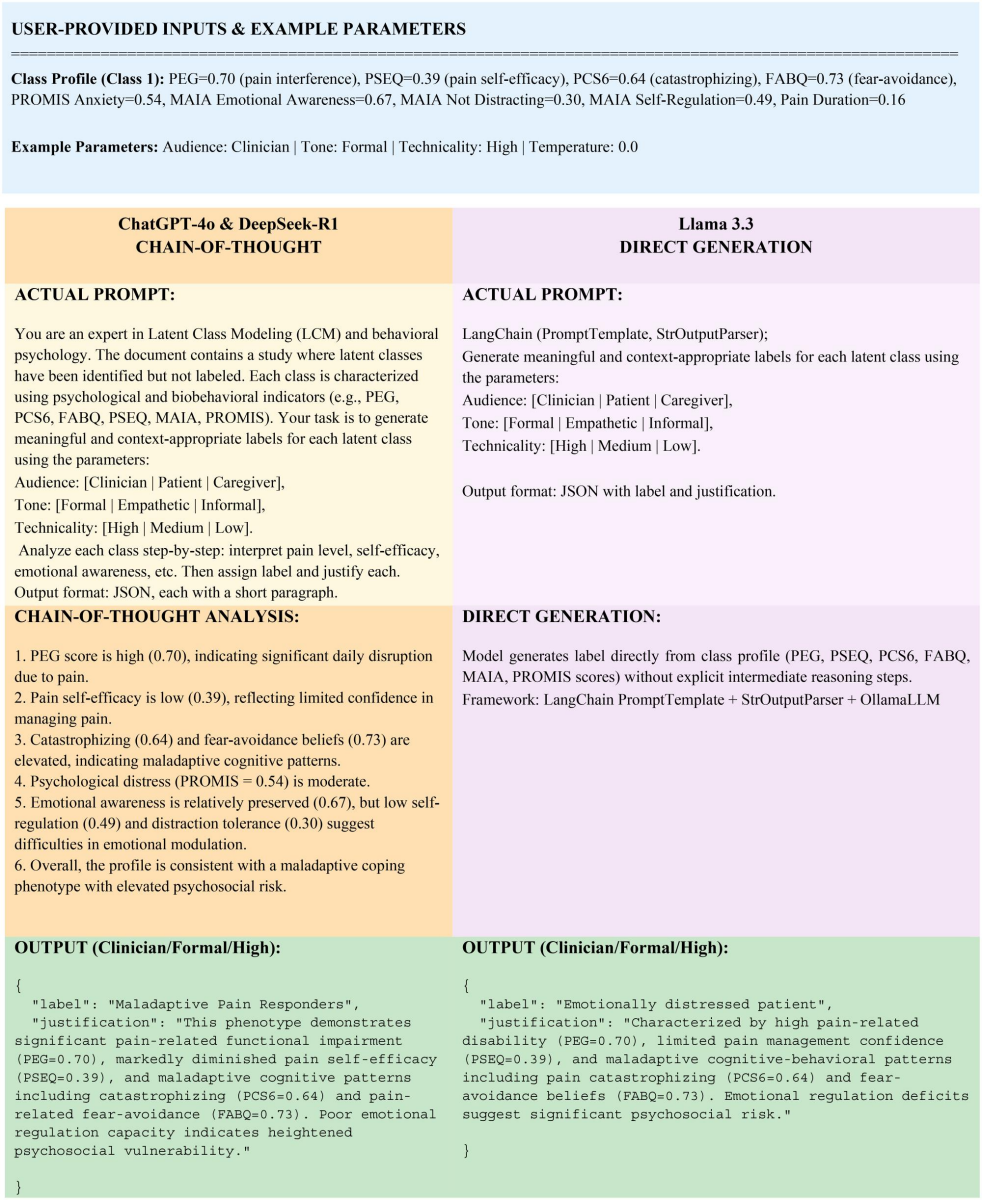
Representative prompting strategies and structured input templates for generating audience-specific labels from latent class profiles.

Overall, this approach was intended to enhance the translation of statistical findings into practical healthcare applications. The overall workflow for generating audience-specific labels from latent-class profiles is shown in Figure 1. This four-step process begins with LCM, proceeds by providing identical inputs to all three LLMs, continues with parallel processing using prompting strategies, and concludes with the generation of labeled phenotypes. Figure 2 presents a representative example of this workflow for a single latent class, illustrating the prompting approaches, model reasoning processes, and the resulting clinical labels and justifications.

## Results

### Sample characteristics

A descriptive summary of the demographic characteristics of the BACKHOME and COMEBACK datasets, respectively, is shown in Table 1. The demographic features of the research population for the BACKHOME and COMEBACK data sets, respectively, are summarized in Table 1. In the BACKHOME dataset, 92.3% of individuals identified as non-Hispanic or Latino; the majority were White (85.8%) and female (68.1%). Participants in the COMEBACK dataset were also White (75.7%) and female (57.1%). There was a modest difference in age between the BACKHOME and COMEBACK participants. The BACKHOME participants had a higher mean BMI than in COMEBACK.

**Table 1:** Socio-demographic characteristics.

Variables	BACKHOME (Train set)	COMEBACK (Validation set)
	(*N* = 3025)	(*N* = 450)
**Age**		
Mean (SD)	56.5 (14.2)	55.6 (15.6)
Median [Min, Max]	58 [20,94]	58.5 [18,91]
Missing^a^	5 (0.16 %)	
**Gender**		
Female	2013 (68.1 %)	257 (57.1 %)
Male	941 (31.8 %)	193 (42.8 %)
**BMI**		
Mean (SD)	29.91 (7.5)	26.76 (5.3)
Median [Min, Max]	28.46 [13.3,71.3]	26.25 [7.8,43.9]
Missing^a^	13 (0.4 %)	12 (2.6 %)
**Education**		
Did not complete High school	6 (0.2 %)	3 (0.6 %)
Some Secondary school (or High School) education	47 (1.5 %)	4 (0.8 %)
High school complete	529 (17.4 %)	46 (10.2 %)
Associate’s or Technical Degree complete	525 (17.3 %)	46 (10.2 %)
College or Baccalaureate Degree complete	948 (31.3 %)	190 (42.2 %)
Doctoral or postgraduate education	970 (32.0 %)	161 (35.7 %)
**Race**		
White	2596 (85.8 %)	341 (75.7 %)
Asian	87 (2.8 %)	45 (10.0 %)
Black or African American	142 (4.6 %)	28 (6.2 %)
Native Hawaiian or Pacific Islander	6 (0.20 %)	4 (0.8 %)
American Indian/Alaska Native	20 (0.6 %)	2 (0.4 %)
More than one race	112 (3.7 %)	12 (2.6 %)
Unknown or not reported	62 (2.05 %)	18 (4.0 %)
**Ethnicity**		
Not Hispanic or Latino	2793 (92.3 %)	392 (87.1 %)
Hispanic or Latino	185 (6.1 %)	51 (11.3 %)
Prefer not to answer or Unknown	47 (1.5 %)	7 (1.5 %)

a Observation with missing values were excluded from the analysis.

### Latent class model profiles

Our previous paper^27^ constructed and detailed the latent class model profiles (see Supplementary Appendix B). Domain experts defined and categorized four classes in that work: Class 1 (“*High Distress and Maladaptive Behaviors*”), Class 2 (“*Resilient and Adaptive Coping*”), Class 3 (“*Intermediate Maladaptive Patterns*”), and Class 4 (“*Emotionally Regulated with High Pain Burden*”). Class 1 was indicated by the highest levels of anxiety/depression, fear avoidance, PCS-6, and PEG scores, indicating a greater propensity for pain and maladaptive avoidance behaviors. In contrast, Class 2 demonstrated lower scores on maladaptive measures, suggesting greater pain self-efficacy and fewer challenges with pain management. Differences across MAIA scales contributed less to class separation, although distinctions between Classes 1 and 2 were evident. Classes 3 and 4 showed intermediate means across several indicators, representing a spectrum of psychological profiles within the population. Class 4, compared to Class 3, presented with higher pain-related scores (PEG), higher fear avoidance, but showed lower emotional awareness and self-regulation. These class profiles define distinct behavioral phenotypes and form the foundation for the automated label generation process with LLMs utilized in the current study.

### Automated audience-adaptive class labeling with LLMs

We used three sophisticated LLMs, ChatGPT-4o, DeepSeek-R1, and Llama 3.3, to automate the tagging of latent classes using a CoT prompting method, building on previously defined latent class profiles. Standardized class profiles, thorough explanations of all psychological and bio-behavioral variables, and clear instructions to create labels with a specific tone and level of sophistication for three audiences: clinicians, patients, and caregivers, were given to each model. The ChatGPT findings are shown in Table 2A. ChatGPT employed a formal tone and high technicality for clinicians, yielding labels such as “High Pain-Maladaptive Coping” and “Resilient-Adaptive Copers,” which focused on behavioral patterns and diagnostic accuracy. On the other hand, patient-focused labels were more empathetic and accessible, including “Stuck in Pain Cycle” and “Managing Well.” ChatGPT generated informal and encouraging labels for caregivers, such as “Stuck in Safety Mode” and “Doing Pretty Well,” emphasizing consolation and useful coping.

**Table 2:** Audience-adapted latent class labels generated by (A) ChatGPT-4o, (B), DeepSeek-R1 and (C) Llama 3.3.

Audience	Tone	Technicality	Class1	Class2	Class3	Class4
**(A)**						
Clinicians	Formal	High	Maladaptive Pain Responders	Resilient Copers	Transitional Responders	Pain-Enduring Copers
Patients	Empathetic	Low	Feeling Overwhelmed by Pain	Staying Strong with Pain	Managing but Sometimes Struggling	Coping Even with High Pain
Caregivers	Informal	Low	Pain and Worry Mix	Doing Well	Finding Their Way	Gets Through with Grit
**(B)**						
Clinicians	Formal	High	High Pain-Maladaptive Coping	Resilient Adaptive Copers	Moderate Pain Intermediate Coping	High Pain Emotionally Aware
Patients	Empathetic	Low	Stuck in Pain Cycle	Managing Well	Mixed Feelings	Pain with Understanding
Caregivers	Informal	Low	Stuck in Safety Mode	Doing Pretty Well	Middle Ground	Thinks It Through
**(C)**						
Clinicians	Formal	High	Emotionally distressed patient	Resilient Pain Manager	Moderate Challenged Individual	Adapting Patient with Resilience
Patients	Empathetic	Low	Challenged by Pain	Resilient Copers	Balanced Mangers	Emotionally Aware Copers
Caregivers	Informal	Low	High Distress Patient	Resilient Pain Manager	Balanced Coping Patient	Steady Progress Patient

The DeepSeek-generated class labels are displayed in Table 2B. For clinicians’ labels, such as “Maladaptive Pain Responders” and “Resilient Copers,” also prioritized clinical clarity and diagnostic language. Patient-focused labels, such as “Feeling Overwhelmed by Pain” and “Staying Strong with Pain,” captured the emotional and experiential aspects of the latent profiles. In contrast, caregiver labels like “Pain and Worry Mix” and “Doing Well” were crafted to acknowledge the challenges and resilience observed by those in a supportive role. Table 2C shows Llama 3.3 audience-specific labeling performed using LangChain’s PromptTemplate and StrOutputParser. For clinicians, Llama 3.3 provided formal, high-technicality descriptors such as “Emotionally Distressed Patient” and “Resilient Pain Manager.” Patient-focused outputs, like “Challenged by Pain” and “Resilient Copers,” emphasized personal experience and emotional nuance. Labels for caregivers, such as “High Distress Patient” and “Steady Progress Patient,” combined a recognition of emotional burden with an optimistic view on adaptation and progress.

Even though the inputs were the same, the three LLMs showed different stylistic trends. Llama highlighted emotional regulation and resilience across all classes, even when describing high-distress presentations (“Emotionally Distressed Patient” vs other models’ “Maladaptive Pain Responders”). DeepSeek prioritized emotional experience and coping persistence, incorporating emotionally resonant language (“Feeling Overwhelmed by Pain,” “Pain and Worry Mix”). ChatGPT made the most granular severity distinctions, using transitional language to capture the range of presentations (“Managing but Sometimes Struggling”). These stylistic variations may be significant when choosing models for specific clinical settings or organizational communication styles, as they reflect different model architectures and training data.

Across all LLMs, the generated class names reliably reflected the core psychological and behavioral characteristics identified by expert manual labeling, such as high distress and maladaptive behaviors, resilience and adaptive coping, or emotional regulation with high pain burden. This conceptual alignment shows how well LLMs interpret latent class profiles and produce audience-specific yet semantically accurate labels.

## Discussion

In this study, we compared three advanced large language models (LLMs), ChatGPT-4o, DeepSeek-R1, and Llama 3.3, for automating the labeling of latent classes using a CoT prompting strategy. Our approach was grounded in previously established latent class profiles,^27^ in which domain experts had identified and categorized four distinct classes: Class 1 (“*High Distress and Maladaptive Behaviors*”), Class 2 (“*Resilient and Adaptive Coping*”), Class 3 (“*Intermediate Maladaptive Patterns*”), and Class 4 (“*Emotionally Regulated with High Pain Burden*”). The models were then prompted to generate class labels while systematically considering the intended audience (clinician, patient, or caregiver), the suitable tone (formal, empathetic, or informal), and the selected level of technicality (high, medium, or low). Analysis of the LLM-generated labels revealed consistent audience adaptation and contextual nuance (Table 2). For clinicians, all models used a formal tone and high technicality, yielding labels that emphasized diagnostic precision and behavioral patterns. For instance, ChatGPT labeled Class 1 as “High Pain-Maladaptive Coping,” while DeepSeek and Llama generated “Maladaptive Pain Responders” and “Emotionally Distressed Patient,” respectively. For patients, each model adopted an empathetic tone with low technicality, resulting in more accessible, supportive labels such as “Stuck in Pain Cycle” (ChatGPT), “Feeling Overwhelmed by Pain” (DeepSeek), and “Challenged by Pain” (Llama) for Class 1. For caregivers, labels were informal and simple, often reflecting practical concerns or encouragement, such as “Stuck in Safety Mode” (ChatGPT), “Pain and Worry Mix” (DeepSeek), and “High Distress Patient” (Llama). It was also clear that the models differed both stylistically and substantively: Llama frequently focused on emotional regulation and resilience, DeepSeek emphasized coping persistence, and ChatGPT outputs tended to provide more thorough gradations between moderate. There was significant conceptual overlap between the labels produced by LLM and the manual expert labels that had previously been developed.^27^ Recent advances in the field have demonstrated the unique capabilities of LLMs for annotation and interpretability tasks. For instance, Törnberg et al^34^ provided compelling evidence that LLMs, particularly GPT-4, substantially enhance text annotation in the social sciences, outperforming both traditional methods and human coders in accuracy, and enabling more nuanced, context-sensitive analysis. Nahum et al^35^ highlighted the promise of LLMs for improving annotation reliability, including using ensemble approaches (“LLM-as-a-judge”) to flag potential label errors in existing datasets systematically. Building on these findings, our work shows that LLMs can also be effectively leveraged for automated class labeling in the health and behavioral sciences. According to the thematic and content analysis results, the LLM pipeline systematically assigned class labels to key bio—behavioral features for every kind of audience. The CoT approach increased transparency since most labels included clear, understandable explanations of the reasoning behind each assignment. This promotes the practical conversion of statistical findings into valuable insights for end users maintaining scientific rigor.

This study has several strengths. First, the LLM-based labeling approach is flexible enough to accommodate changing data landscapes since labels can be produced quickly for any new class solution or dataset without recurring expert consensus. Due to the high degree of adaptation of the framework, labels can be consistently and reproducibly adapted to different audiences and communication contexts using standardized output formats. The method also makes it easier to update and improve label sets in response to new clinical or scientific requirements by enabling quick prototyping and iterative development. Other significant advantages of CoT prompting are transparency and interpretability, which enable more candid conversations with stakeholders and let users comprehend the rationale behind each label. Further expanding the usefulness of latent class modeling, the LLMs’ structured, machine-readable outputs facilitate smooth integration with clinical decision support systems, patient education resources, and digital health platforms. However, this study also has limitations. Due to the biases in their training data, LLMs may not produce results that accurately reflect all patient populations’ contextual or cultural nuances. Importantly, this study did not include direct feedback from end-users (clinicians, patients, or caregivers) on the interpretability and utility of the generated labels, a key area for future research.

Further research is needed to test these findings in different clinical settings, assess user acceptance and comprehension, and enhance prompt engineering methods to maximize contextual fit and impact. Direct engagement with end-users is essential before broader clinical deployment. Future work should include cognitive interviews with patients to evaluate label clarity and personal relevance, focus groups with clinicians (orthopedic surgeons, pain specialists, physicians) to evaluate diagnostic accuracy and clinical utility, and brief implementation studies in clinical settings to assess real-world adoption and impact on clinical decision-making. For more generalizability, the framework must be expanded to encompass non-English speaking populations and a greater range of healthcare scenarios. LLM-generated labels’ contextual sensitivity and clarity will also need further improvement through ongoing model updates and prompt design enhancements. Finally, advancing clinical decision assistance and tailored communication at scale may be possible by integrating LLMs’ labeling pipelines into digital health infrastructures.

## Conclusion

This study shows that LLMs can efficiently and effectively generate interpretable, audience-specific labels for clinicians, patients, and caregivers for latent class analysis in health and behavioral research. Our approach bridges the gap between statistical modeling and practical, stakeholder-centered communication. Embracing LLM-based labeling frameworks improves the clarity, consistency, and scalability of latent class analysis while decreasing time to obtain expert consensus. These results highlight the possibility of integrating LLM-driven labeling into research and clinical practice, helping to achieve more transparent knowledge translation, improved decision-making, and personalized care.

## Supplementary Material

ooag058_Supplementary_Data

## Data Availability

The data that support the findings of this study are available in the Dryad Digital Repository (https://doi.org/10.5061/dryad.1jwstqk9d). The original datasets are available through the Vivli repository (https://doi.org/10.25934/PR00010820 and https://doi.org/10.25934/PR00010819) and require approval for access.
