# Supplementary material for: Large language models for automated and audience-tailored labeling of latent classes
Source: JAMIA Open. 2026 Apr 28;9(2):ooag058. doi: 10.1093/jamiaopen/ooag058 (PMC13127414; doi:10.1093/jamiaopen/ooag058)
Supplement: ooag058_Supplementary_Data [file ooag058_supplementary_data.docx]

**Appendices**

**A: Data Dictionary**

Below, we will provide a more detailed explanation of the variables used in this study.

**PEG:** The PEG Score is calculated from the mean of the following three questions each range 0-10:

- What number best describes your pain on average, in the past week?
- What number best describes how, during the past week, pain has interfered with your enjoyment of life?
- What number best describes how, during the past week, pain has interfered with your general activity?

(0 = no pain; 10 = worst pain imaginable)

**PROMIS Pain Interference:**  Score is calculated as the sum of the following 4 values each ranged 1-5: In the past 7 days, how much did pain interfere with your:

- day to day activities?
- work around the home?
- ability to participate in social activities?
- household chores?

Where 1 corresponds to ‘not at all’; 2 corresponds to ‘a little bit‘; 3 corresponds to ‘somewhat‘;4 corresponds to ‘quite a bit‘ and 5 corresponds to ‘very much‘.

**Fear Avoidance:** This is the FABQ-P (Fear Avoidance Beliefs with Physical Activity) score, which is calculated as 4 times the mean of their answers to the following questions:

- Physical activity makes my pain worse
- Physical activity might harm my back
- I should not do physical activities which (might) make my pain worse
- I cannot do physical activities which might make my pain worse

Where responses are on a scale from 0-6 where 0 means completely disagree and 6 is completely agree, thus a higher score means more fear.

**Duration Low Back Pain**: duration of low back pain (total years).

**Average Anxiety and Depression**: T-scores from PROMIS Anxiety and Depression scales.

- PROMIS Depression: (range:41.0-79.4, higher=more depressed)
- PROMIS Anxiety : (range 40.3-83.1; higher=more severe anxiety)

**Not Distracting**: This is the MAIA-SF (Multi-dimensional Assessment of Interoceptive Awareness, v2) – Not- Distracting Sub-Score. Participants are asked to answer the following question, Please indicate how often each statement applies to you generally in daily life:

- I ignore physical tension or discomfort until they become more severe.
- I distract myself from sensations of discomfort.
- When I feel pain or discomfort, I try to power through it.
- I try to ignore pain.
- I push feelings of discomfort away by focusing on something.
- When I feel unpleasant body sensations, I occupy myself with something else, so I don’t have to feel them.

Where responses are on a scale from 0-5 where 0 means never and 5 means always.

The non-distracting sub-score is then calculated as the mean of 5 minus each of their responses, thus a higher score means less distracting.

**Emotion Aware:** This is the MAIA-SF Emotional Awareness Sub-Scale score which is the mean of their answers to the following questions:

• I notice how my body changes when I am angry.

• When something is wrong in my life I can feel it in my body.

• I notice that my body feels different after a peaceful experience.

• I notice that my breathing becomes free and easy when I feel comfortable.

• I notice how my body changes when I feel happy / joyful.

where responses are on a scale from 0-5 where 0 means never and 5 means always, thus a higher score means more awareness.

**Self Regulation:** This is the MAIA-SF Self-Regulation Sub-Scale which is the mean of their answers to the following questions:

• When I feel overwhelmed, I can find a calm place inside.

• When I bring awareness to my body I feel a sense of calm.

• I can use my breath to reduce tension.

• When I am caught up in thoughts, I can calm my mind by focusing on my body/breathing.

where responses are on a scale from 0-5 where 0 means never and 5 means always, thus a higher score means more self-regulation.

**Pain Catastrophizing**: This is the PCS-6 score, which is calculated as 3 times the mean of helplessness sub-score, magnification sub-score, and rumination sub-score.

The helplessness sub-score is calculated as the mean of their answers to the following questions:

- It’s awful and I feel that it overwhelms me
- I feel I can’t stand it anymore

The magnification sub-score is calculated as the mean of their answers to the following questions:

- I become afraid that the pain will get worse
- I keep thinking about how much it hurts

The rumination sub-score is calculated as the mean of their answers to the following questions:

- I keep thinking about how badly I want the pain to stop
- I wonder whether something serious may happen

For all questions, responses are on a scale of 0-4 where:

- 0: Not at all
- 1: To a slight degree
- 2: To a moderate degree
- 3: To a great degree
- 4: All the time

Thus, a higher score means more catastrophizing.

**Self-Efficacy:** This is the PSEQ-4 (Pain Self-Efficacy) score, which is calculated as 4 times the mean of their answers to the following questions:

- I can cope with my pain in most situations.
- I can still do many of the things I enjoy doing, such as hobbies or leisure activity, despite pain
- I can still accomplish most of my goals in life, despite the pain.
- I can live a normal lifestyle, despite the pain.

Where responses were originally on a scale of 1-6 with 1 meaning not at all confident and 6 meaning completely confident. These responses were then mapped to the following values for calculation:

1: 0

2: 1.2

3: 2.4

4: 3.6

5: 4.8

6: 6

# **Appendix B: Normalized Class Profiles for BACKHOME (Train) and COMEBACK (Test) Sets**

## **BACKHOME (Train Set)**

| **Feature** | **Class_1 (n = 701)** | **Class_2 (n = 413)** | **Class_3 (n = 893)** | **Class_4 (n = 947)** |
| --- | --- | --- | --- | --- |
| PAIN SELF EFFICACY SCORE | 0.39 | 0.87 | 0.68 | 0.66 |
| PAIN CATASTROPHIZING SCALE | 0.64 | 0.16 | 0.35 | 0.35 |
| PEG | 0.70 | 0.23 | 0.42 | 0.45 |
| FEAR AVOIDANCE SCORE | 0.73 | 0.30 | 0.44 | 0.49 |
| AVERAGE PROMIS ANXIETY/DEPRESSION | 0.54 | 0.05 | 0.30 | 0.29 |
| MAIA2 EMOTIONAL AWARENESS SCALE | 0.67 | 0.49 | 0.59 | 0.56 |
| MAIA2 NOT DISTRACTING SCALE | 0.30 | 0.38 | 0.35 | 0.34 |
| MAIA2 SELF REGULATION SCALE | 0.49 | 0.59 | 0.54 | 0.53 |
| DURATION LOW BACK PAIN | 0.16 | 0.18 | 0.03 | 0.26 |

**COMEBACK (Test Set)**

| **Feature** | **Class_1 (n = 127)** | **Class_2 (n = 108)** | **Class_3 (n = 95)** | **Class_4 (n = 68)** |
| --- | --- | --- | --- | --- |
| PAIN SELF EFFICACY SCORE | 0.39 | 0.79 | 0.68 | 0.66 |
| PAIN CATASTROPHIZING SCALE | 0.70 | 0.16 | 0.35 | 0.35 |
| PEG | 0.68 | 0.20 | 0.42 | 0.45 |
| FEAR AVOIDANCE SCORE | 0.73 | 0.29 | 0.44 | 0.49 |
| AVERAGE PROMIS ANXIETY/DEPRESSION | 0.30 | 0.08 | 0.24 | 0.40 |
| MAIA2 EMOTIONAL AWARENESS SCALE | 0.53 | 0.59 | 0.69 | 0.60 |
| MAIA2 NOT DISTRACTING SCALE | 0.40 | 0.38 | 0.35 | 0.34 |
| MAIA2 SELF REGULATION SCALE | 0.52 | 0.59 | 0.60 | 0.53 |
| DURATION LOW BACK PAIN | 0.20 | 0.15 | 0.06 | 0.30 |
